# Species Delimitation and Interspecific Relationships of the Genus *Orychophragmus* (Brassicaceae) Inferred from Whole Chloroplast Genomes

**DOI:** 10.3389/fpls.2016.01826

**Published:** 2016-12-06

**Authors:** Huan Hu, Quanjun Hu, Ihsan A. Al-Shehbaz, Xin Luo, Tingting Zeng, Xinyi Guo, Jianquan Liu

**Affiliations:** ^1^MOE Key Laboratory for Bio-Resources and Eco-Environment, College of Life Science, Sichuan UniversityChengdu, China; ^2^Missouri Botanical GardenSt. Louis, MO, USA

**Keywords:** *Orychophragmus*, Brassicaceae, chloroplast genome, phylogenetic relationship, species delimitation

## Abstract

Genetic variations from few chloroplast DNA fragments show lower discriminatory power in the delimitation of closely related species and less resolution ability in discerning interspecific relationships than from nrITS. Here we use *Orychophragmus* (Brassicaceae) as a model system to test the hypothesis that the whole chloroplast genomes (plastomes), with accumulation of more variations despite the slow evolution, can overcome these weaknesses. We used Illumina sequencing technology via a reference-guided assembly to construct complete plastomes of 17 individuals from six putatively assumed species in the genus. All plastomes are highly conserved in genome structure, gene order, and orientation, and they are around 153 kb in length and contain 113 unique genes. However, nucleotide variations are quite substantial to support the delimitation of all sampled species and to resolve interspecific relationships with high statistical supports. As expected, the estimated divergences between major clades and species are lower than those estimated from nrITS probably due to the slow substitution rate of the plastomes. However, the plastome and nrITS phylogenies were contradictory in the placements of most species, thus suggesting that these species may have experienced complex non-bifurcating evolutions with incomplete lineage sorting and/or hybrid introgressions. Overall, our case study highlights the importance of using plastomes to examine species boundaries and establish an independent phylogeny to infer the speciation history of plants.

## Introduction

It is rather difficult to delimit recently diverged species and construct their interspecific relationships because of insufficient informative variations of sampled DNA fragments (Schluter, 2000; Arnold, 2006). The genome-scale sequence variations were found to increase the phylogenetic resolutions of both high- and low-taxonomic groups (e.g., Yoder et al., 2013; Lamichhaney et al., 2015). It is still expensive to collect nuclear genome variations between species for most none-model genera without the reference genome. However, chloroplast genomes (plastome) are relatively easy to be assembled to examine interspecific relationships for phylogenetic analyses, especially in addressing unresolved relationship at low taxonomic levels (Wu et al., 2010; Nock et al., 2011; Yang et al., 2013; Huang et al., 2014; Carbonell-Caballero et al., 2015). Plastomes are haploid with maternal inheritance in most angiosperms (Corriveau and Coleman, 1988; Zhang and Liu, 2003; Hagemann, 2004) and are highly conservative in gene order and genome structure with rare recombinations (Jansen et al., 2007; Moore et al., 2010). In this study, we aimed to examine species delimitation and interspecific relationships in *Orychophragmus* through assembling chloroplast genomes of multiple individuals of tentatively delimited species (Hu H. et al., 2015).

*Orychophragmus* is a small genus in the mustard family (Brassicaceae, Cruciferae) distributed in northern, central, and southeastern China (Zhou et al., 2001). Its plants have been widely cultivated as ornamentals, vegetables, or source of seed oil (Sun et al., 2011). Despite controversial species delimitations in the genus (Zhou, 1987; Tan et al., 1998; Al-Shehbaz and Yang, 2000; Zhou et al., 2001; Wu and Zhao, 2003; Sun et al., 2012), our recent study based on nuclear (nr) ITS sequence variations suggested the recognition of seven species (Hu H. et al., 2015). *Orychophragmus* is sister to *Sinalliaria*, which is a genus endemic to China with one (Zhou et al., 2014) or two independent species (Hu H. et al., 2015). Nuclear ITS sequence variations support the recognition of seven species and strongly resolve their interspecific relationships, but four DNA fragments (*mat*K, *rbc*L, *trn*H-*psb*A, and *trn*L-F) of the chloroplast genome failed to do so (Hu H. et al., 2015). It was hypothesized that this was caused by slow rates of chloroplast DNA evolution, frequent introgression, and incomplete lineage sorting. In order to test these hypotheses, we assembled 17 plastomes from six species of the genus using next-generation Illumina genome-analyzer platform with a guided-reference plastome. We aimed to address the following questions: (1) could the plastome sequence variations confirm the species delimitation obtained by the ITS dataset? (2) are interspecific relationships well resolved with high statistical supports? (3) if so, are phylogenetic relationships based on the plastome sequence variations consistent or inconsistent with those obtained from the nuclear ITS dataset?

## Materials and methods

### Plant materials

We used at least two individuals from two different populations for each of six species, except the recently extinct *Orychophragmus ziguiensis*. In total, 17 individuals from 17 populations were sampled (see Table S1), and fresh leaves of each individual were immediately dried in silica gel for DNA extraction. For the final analyses, we downloaded five complete plastomes from five populations of two species of *Sinalliara* (Zeng et al., 2016). We also included the plastomes of *Brassica napus* L., *B. juncea* (L.) Czern., *Eutrema heterophyllum* (W. W. Sm) H. Hara, *Lobularia maritima* (L.) Desv., *Capsella grandiflora* (Fauché & Chaub.) Boiss., *Arabidopsis thaliana* (L.) Heynh., and *Aethionema grandiflorum* Boiss. and Hohen. That we downloaded from GenBank (Genbank accessions see in Table S1). The plastome of *Carica papaya* L. was used as the outgroup for phylogenetic analyses.

### DNA extraction and plastome sequencing

Total genomic DNA was extracted from 20 mg silica gel-dried leaves by using a modified 2 × cetyltrimethylammonium bromide (CTAB) procedure (Doyle, 1987). The library construction and sequencing were finished at the Beijing Novogene bio Mdt InfoTech Ltd (Beijing, China). The qualified and purified DNA samples were randomly fragmented with a Covaris sonication device. The DNA fragments were end-repaired, phosphorylated, and A-tailed. Adapters were then ligated with index adapters. The ligated fragments were amplified for library construction. The qualified libraries were applied to an Illumina flowcell for cBOT cluster generation. Sequencing was performed on an Illumina MiSeq instrument.

### Plastome assembly and annotation

The raw reads for all samples contained a few adapter-related paired reads. Reads with over 10% containing N or with low quality (Q <= 5) were trimmed to acquire clean reads to ensure the high-quality following analysis. All of the clean reads were initially mapped to all published Brassicacae chloroplast genomes (29 species) using BWA v.0.7.12 (Li and Durbin, 2009) and SAMtools v.1.2 (Li et al., 2009). We then applied Velvet v.1.2.10 (Zerbino and Birney, 2008) to assemble these reads into the complete plastid genomes, and gaps were filled with GapCloser v.1.12 (http://soap.genomics.org.cn/index.html). We finally annotated the chloroplast genomes using Plann v.1.1.2 (Huang and Cronk, 2015) and manually corrected for start and stop codons and for intron/exon boundaries to match gene predictions with Geneious v.R.8.1.4 (Kearse et al., 2012) and Sequin v.15.10 (http://www.ncbi.nlm.nih.gov/Sequin/) based on *Arabidopsis thaliana* chloroplast genome as a reference annotation. The visual images about annotation information were generated by OGDRAW v.1.1 (http://ogdraw.mpimp-golm.mpg.de/) (Lohse et al., 2013). Full alignments with annotation information were plotted using the mVISTA (Mayor et al., 2000). All plastomes are reported here for the first time and were submitted to GenBank with the accession numbers of KX364399 and from KX756547 to KX756551.

### Phylogenetic analyses

All plastome sequences were aligned using MAFFT v.7 (Katoh and Standley, 2013) and adjusted manually where necessary using MEGA v.6 (Tamura et al., 2013). Phylogenetic analyses were performed by using the whole plastome data and the aligned data including only the hotspot mutation regions. We used JModeltest v.2.1.1 (Posada, 2008; Darriba et al., 2012) to select the most appropriate nucleotide substitution model and parameter settings for Bayesian analyses based on Bayesian Information Criterion (BIC) (the best-fit model chosen was TVM+I+G). To avoid the potential heterogeneity within whole plastome sequences resulting in un-reliable phylogenetic reconstruction (Arbiza et al., 2011; Zhong et al., 2011; Sun et al., 2016), we tested evolutionary model for each plastome section (all full-length sequence was divided into three sections: LSC, SSC, and IRs or two sections: coding region and non-coding region). All plastome sections followed TVM+I+G model based on BIC or AIC (Table S2). We employed RAxML v.8.1.24 (Stamatakis, 2014) to reconstruct Maximum Likelihood (ML) tree with 500 bootstraps under the GTRGAMMAI substitution model. We also used MrBayes v.3.2.2 (Ronquist and Huelsenbeck, 2003; Ronquist et al., 2012) for the Bayesian inference analyses. MrBayes was run for 1,000,000 generations, sampling and printing every 100 generations. We conducted two independent Markov Chain Monte Carlo (MCMC) runs with four chains (one cold and three hot). We estimated branch supports from 500 ML bootstrap values (BS) and from the posterior probabilities (PP) of Bayesian trees after a 50% “burn-in.”

### Estimate of divergence time

We used the whole plastome data and the hotspot mutation region data to estimate divergence time. Due to the lack of fossil record, we used the average substitution rate 0.051952 ± 0.000537 × 10^−8^ substitutions per site per year or two fixed calibrations (23.5 million years ago (Ma) between the *Arabidopsis* clade vs. the sister clade and 20.85 Ma between the *Lobularia* subclade vs. the sister subclade) estimated from the calibrated plastome phylogeny of Brassicaceae (Hohmann et al., 2015) to estimate the species divergence within the genus. The Bayesian dating analysis was performed with a relaxed clock approach using BEAST v.2.4.0 (Bouckaert et al., 2014). BEAST runs were conducted by choosing the general time-reversible model GTR+I+G and getting relative parameter settings (TVM+I+G) from JModeltest software. For each analysis, we ran 100,000,000 generations of MCMC run, sampling parameters every 10,000 generations, using a lognormal relaxed clock model (Drummond et al., 2006) under a Yule speciation tree prior with the substitution rate. The convergence of the MCMC searches and the effective samples size (ESS) of the posterior probability were in most cases >200, and always >150 for every estimated parameter were checked in Tracer v.1.6 (Rambaut et al., 2014). Two replicates were combined by removing 25% as burn-in using LogCombiner v.2.4.0 (Bouckaert et al., 2014). TreeAnnotator v.2.4.0 (Bouckaert et al., 2014) was used to produce maximum clade credibility trees (MCCT) from the post-burn-in trees and to determine the 95% posterior density of ages for all nodes in the tree by setting burning-in of 25% and a posterior probability limit of 0.5. The final tree was visualized in FigTree v.1.4.2 (http://tree.bio.ed.ac.uk/software/figtree/).

## Results

### Conservative features of *Orychophragmus* plastomes

**Figure 1:**
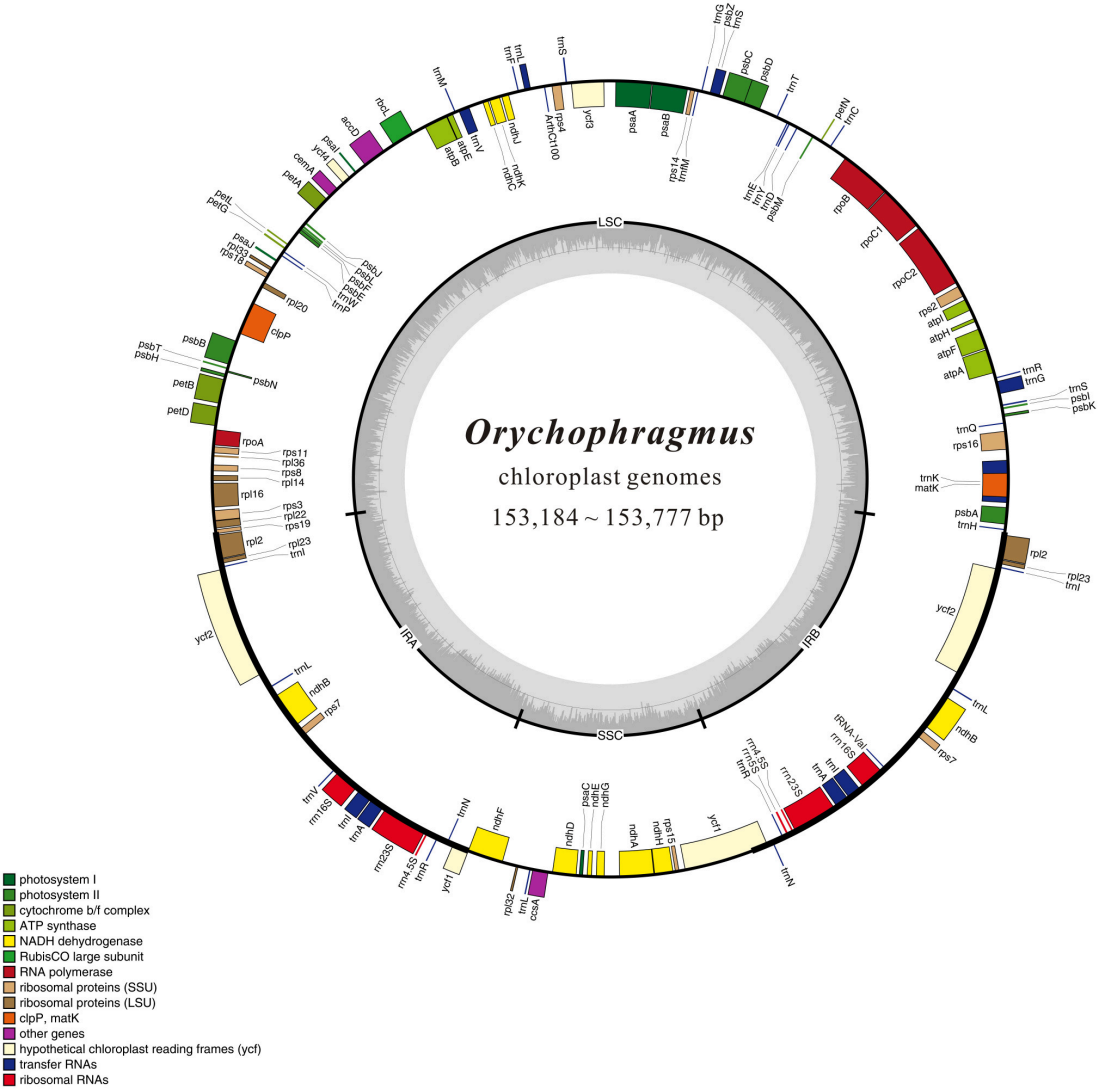
**Gene map of the ***Orychophragmus*** chloroplast genomes**. Genes shown outside of the map circle are transcribed clockwise, while those drawn inside are transcribed counterclockwise. Genes belonging to different functional groups were color-coded. The innermost darker gray corresponds to GC while the lighter gray corresponds to AT content of the chloroplast genomes.

**Figure 2:**
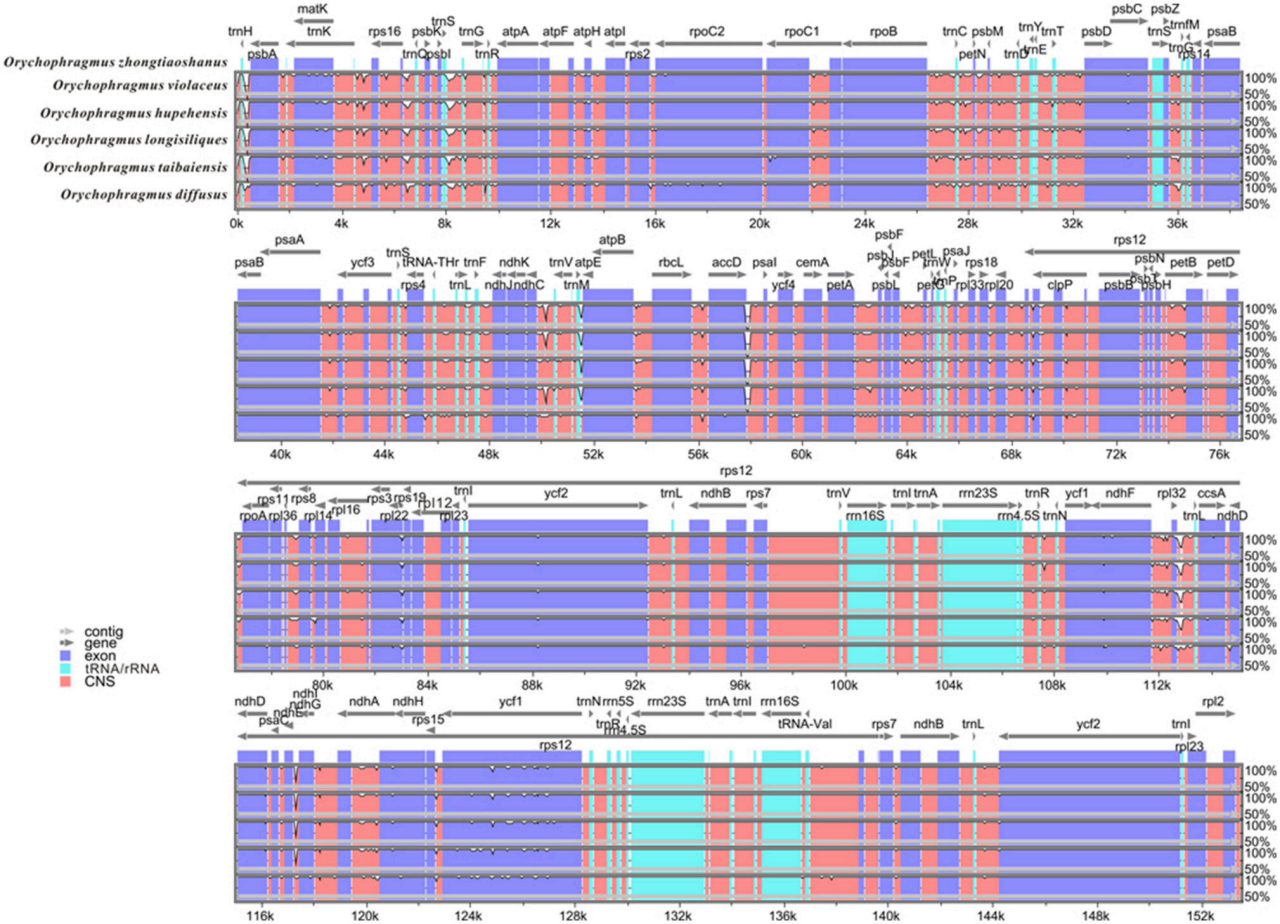
**Visualization of alignment of the six ***Orychophragmus*** chloroplast genome sequences**. VISTA-based identity plots showing sequence identity between six sequenced cp genomes of *Orychophragmus*. Gray arrows above the alignment indicate genes with their orientation. A cut-off of 70% identity was used for the plots, and the Y-scale represents the percent identity (50–100%), the X-axis represents the coordinate in the chloroplast genome. Genome regions are color-coded as protein coding, rRNA coding, tRNA coding or conserved noncoding sequences.

1.26 ~ 1.97 G clean base of each individual of six species of *Orychophragmus* obtained from Next Generation Sequencing (NGS, Table 1). And plastomes recovered here are 153,182 ~ 153,777 bp in size, consisting of a pair of inverted repeats (IRa and IRb) of 26,222 ~ 26,255 bp separated by large and small copy (LSC and SSC) regions of 83,057 ~ 83,456 and 17,676 ~ 17,813 bp, respectively (Table 1). The plastomes consistently contained 129 genes, including 85 protein-coding genes (79 PCG species), 37 tRNA genes (30 tRNA species), and 7 ribosomal RNA genes (4 rRNA species) (Figure 1). Most genes occurred in a single copy, while 16 were duplicated on the IR regions, including 3 rRNA (4.5S, 16S, and 23S rRNA), 7 tRNA, and 6 PCG species (*rpl*2, *rpl*23, *ycf* 2, *ndh*B, *rps*7, and *ycf* 1). The *rps*12 gene was a unique trans-spliced gene with three exons. The *rps*19 gene was located in the boundary regions between LSC/IRb, and the *ndh*F gene was situated in the boundary regions between IRb/SSC. The gene *ycf* 1 was crossed at the junction of IRb/SSC and SSC/IRa, leading to incomplete duplication of the protein-coding gene within IRs (Figure 1). The overall GC contents of cpDNA ranged from 36.28 to 36.35% (Table 1), suggesting that the AT-rich contents of this genus are similar to other Brassicaceae plastid genomes sequenced so far (Hu S. L. et al., 2015). In general, the genome features of six species were found to be quite similar in gene content, gene order, introns, intergenic spacers, and AT content. The overall sequences identity of 16 plastomes was visualized using the mVISTA tool (Mayor et al., 2000) based on the annotation of one of them (*O. taibaiensis*) as a reference with LAGAN mode. The sequences identity was 98% between all plastomes. Moderate genetic divergences were detected, and the most divergent regions were located in the intergenic spacers while nine divergence hotspot regions were identified (Figure 2).

**Table 1:** **Chloroplast assemble and annotation information for six species of ***Orychophragmus*****.

	**No. of populations**	**Raw reads**	**Clean reads**	**Raw base (G)**	**Clean base (G)**	**Entire plastid size (bp)**	**LSC**	**SSC**	**IR (two copies)**	**Overall GC content (%)**	**Number of genes (different/total)**	**Number of proten-coding genes**	**Number of rRNA genes**	**Number of tRNA genes**	**Number of different genes duplicated by IR**
***O. violaceus***	13,001	4,283,925	4,245,608	1.28	1.27	153,234	83,070	17,704	26,230	36.347	113/129	85	7	37	16
	13,032	4,424,055	4,374,950	1.33	1.31	153,225	83,063	17,700	26,231	36.345	113/129	85	7	37	16
	13,034	4,534,517	4,510,599	1.36	1.35	153,238	83,073	17,703	26,231	36.352	113/129	85	7	37	16
***O. zhongtiaoshanus***	14,010	4,349,059	4,192,589	1.3	1.26	153,440	83,225	17,757	26,229	36.278	113/129	85	7	37	16
	13,028	5,230,868	5,215,393	1.57	1.56	153,442	83,224	17,762	26,228	36.283	113/129	85	7	37	16
	13,027	4,830,075	4,816,712	1.45	1.45	153,416	83,215	17,757	26,222	36.288	113/129	85	7	37	16
***O. diffuses***	13,017	5,067,862	5,029,109	1.52	1.51	153,777	83,456	17,811	26,255	36.289	113/129	85	7	37	16
	13,014	6,595,002	6,570,487	1.98	1.97	153,765	83,444	17,813	26,254	36.285	113/129	85	7	37	16
	13,016	6,084,975	6,052,878	1.82	1.82	153,775	83,456	17,813	26,253	36.289	113/129	85	7	37	16
***O. longisiliqus***	13,006	5,018,488	4,998,911	1.51	1.5	153,365	83,212	17,687	26,233	36.322	113/129	85	7	37	16
	13,019	4,794,575	4,748,735	1.44	1.43	153,354	83,208	17,680	26,233	36.331	113/129	85	7	37	16
	14,003	4,266,953	4,234,374	1.28	1.27	153,350	83,204	17,682	26,232	36.326	113/129	85	7	37	16
***O. hupehensis***	13,022	5,413,578	5,355,940	1.62	1.61	153,184	83,060	17,676	26,224	36.352	113/129	85	7	37	16
	13,021	4,557,317	4,502,153	1.37	1.35	153,182	83,057	17,677	26,224	36.350	113/129	85	7	37	16
***O. taibaiensis***	14,012	4,560,113	4,542,958	1.37	1.36	153,255	83,106	17,683	26,233	36.339	113/129	85	7	37	16
	13,024	5,958,515	5,920,112	1.79	1.78	153,259	83,107	17,686	26,233	36.338	113/129	85	7	37	16
	13,025	5,264,136	5,229,983	1.58	1.57	153,262	83,110	17,686	26,233	36.356	113/129	85	7	37	16

### Phylogenetic analyses and divergence estimations based on plastome sequence variations

**Figure 3:**
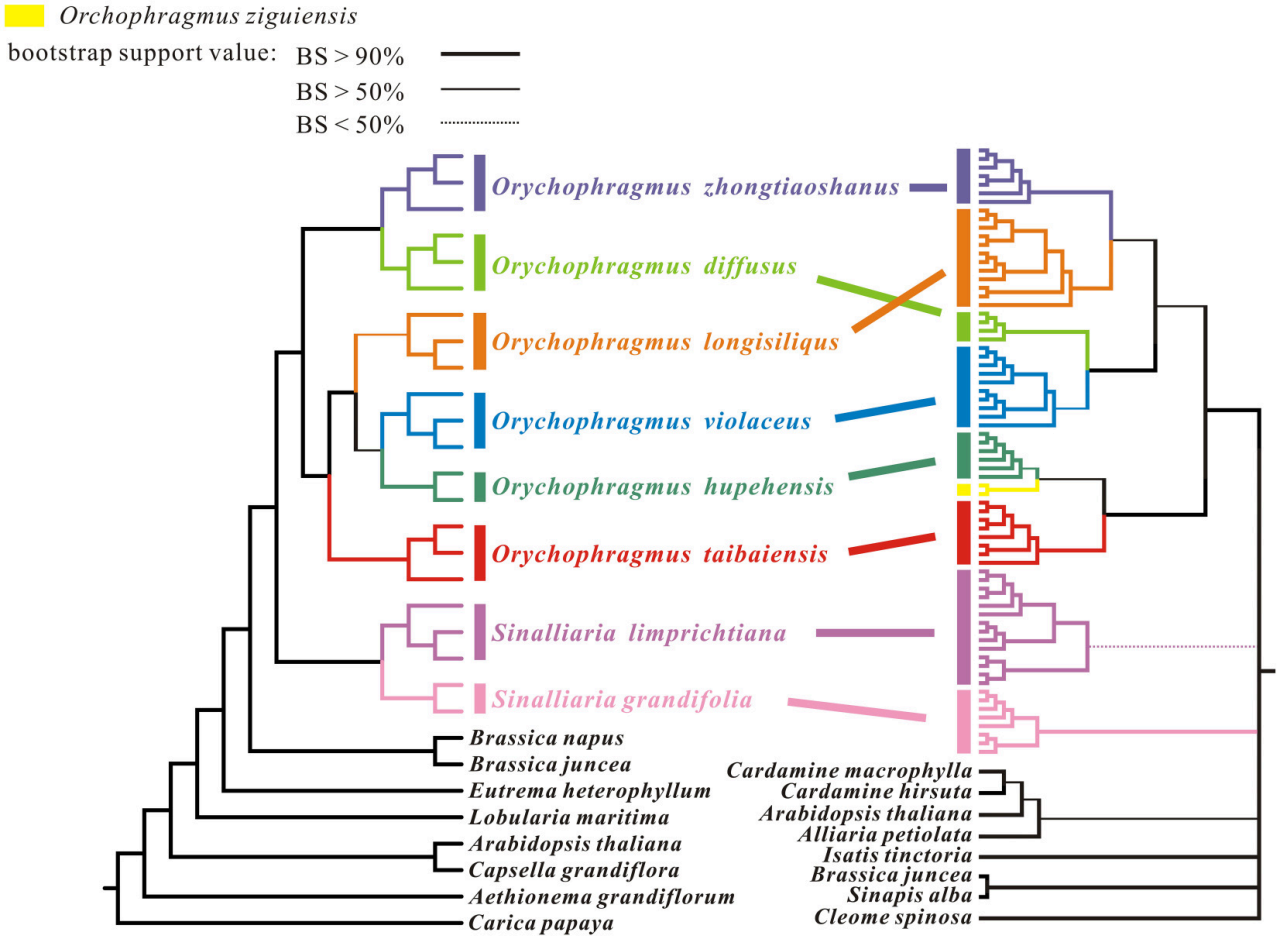
**Maximum likelihood tree based on analyses of whole chloroplast genome sequences for 17 ***Orychophragmus*** individuals and of unique nrITS ribotype sequences**. The left tree is topologically congruent with the Bayesian consensus tree. Statistical support from maximum likelihood with different values were shown as different line forms. The different taxa of *Orychophragmus* and *Sinalliaria* are marked by different colors. Individual number is given after original species name. The right-hand tree was cited from our previous study (Hu H. et al., 2015).

Phylogenetic trees were reconstructed by RAxML and Mrbayse softwares, rooted by the outgroup *Carica papaya*. The ML tree was congruent with the Bayesian consensus tree in the phylogenetic topologies based on the whole plastome data, although statistical supports (BP and PP) were different in some clades or subclades (Figure 3). All posterior probabilities (PP) were higher than bootstrap supports (BP). All sampled individuals of each species were found to cluster together as one monophyletic lineage, although the BP support of *Orychophragmus longisiliqus* was lower than 90%, but larger than 50%. Six species clustered into two clades: one consisted of *O. zhongtiaoshanus* and *O. diffusus*, while the other comprised the remaining four species. In the four-species clade, *O. violaceus* was sister to *O. hupehensis* and together sister to *O. longisiliqus*, whereas *O. taibaiensis* formed an isolated subclade. Phylogenetic analyses of the hotspot mutation regions produced the similar tree topologies, but relationships between species or subclades of the genus *Orychophragmus* remained unsolved (Figure S1).

**Figure 4:**
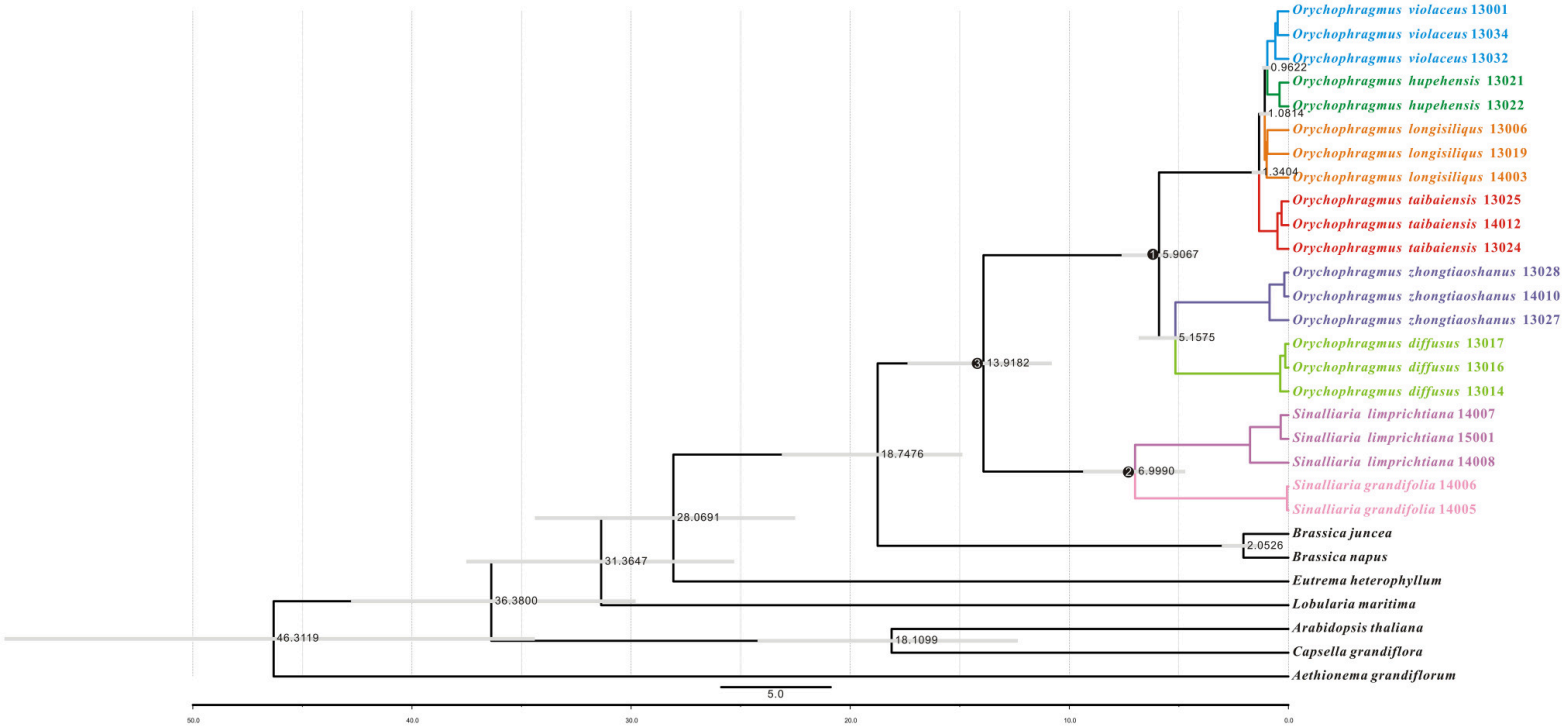
**Divergence time estimates using the average substitution rate based on the whole chloroplast genome sequences**. Divergence times of species based on uncorrelated relaxed clock method, using a substitution rate of 5.1952E-4 per base per million years calculated by BEAST program over the whole chloroplast genome sequences. The legend describes the divergence time in million years, and the gray boxes represent the 95% highest probability density of divergence times. In addition, three major nodes were used for divergence comparisons in the Table S3.

We further estimated divergence time of the genus and among its species using the general plastome substitution rate (Figure 4) and two secondary calibration points (Figure S2). Divergence times estimated by substitution rate the major nodes were general older than times from the secondary calibration points. For instance, the divergence between *Orychophragmus* and sister genus *Sinalliaria* was dated to about 13.92 million years ago (Mya) using the average rate, while this divergence was four million years younger by using two calibration points (Figure S2 and Table S3). In the similar way, the first divergence between the two major clades of *Orychophragmus* was calculated to have occurred around 5.91 or 3.72, while the divergence between the subclades and species ranged from 0.96~0.59 to 5.16~3.25 Mya (Figure 4 and Figure S2; Table S3). As expected, all divergence times of three major nodes (Figure 4) estimated based only on the hotspot mutation regions and the average substation rate were much older (Table S3) than those based on the whole plastome dataset because of the more accumulated divergence. However, when two assumed diverged points were adopted, the divergences of the major nodes were estimated to be similar to those based on the whole plastomes (Table S3).

## Discussion

The plastomes of land plants are known to be highly conserved in genome structure, gene order, and gene content with a quardripartite structure and two copies of large inverted repeat (IR) separating two regions (large single-copy region LSC and small single-copy region SSC) (Raubeson and Jansen, 2005; Jansen and Ruhlman, 2012). Most plastomes are circular and ranged from 120 to 160 kb in length, with 110 to 130 different genes, including 70 (gymnosperms) to 88 (liverworts) protein-coding genes mostly involved in photosynthesis or gene expression, and 33 (most eudicots) to 35 (liverworts) structure RNA genes (Raubeson and Jansen, 2005; Bock, 2007; Wicke et al., 2011). The markers (or DNA fragments) based on sequence variations of plastomes have been widely used to examine population genetic structure, delimit species boundaries, and construct phylogeny due to their high-copy number (as many as 1000 per cell), easy amplification, and relative conservation of all targeted regions (Raubeson and Jansen, 2005; Wicke et al., 2011). In addition, sequence variations of total plastome was found to provide higher resolution in constructing plant phylogenies than few DNA fragments (Parks et al., 2009).

In this study, 17 plastomes for six species of *Orychophragmus* were assembled for the first time, and all have typical quadripartite structure, as in most angiosperms, including LSC, SSC, and a pair of IRa and IRb (Palmer, 1991). The overall sequence identity of all plastomes was high (around 98%), and the divergence between them, especially the insertions-deletions (INDELS), commonly occurred in the intergenic spacers. The nine intergenic spacers of *trn*H-*psb*A, *atp*I-*rps*2, *trn*M-*atp*E, *atp*B-*rbc*L, *ndh*C-*trn*V, *acc*D-*psa*I, *pet*B-*pet*D, *rpl*23-*trn*L, and *ndh*E-*ndh*G were identified as the divergence hotspots of sequence similarities below 50% (Figure 2), which would be useful if these regions were developed as potential molecular markers for identification of the different species units for the genus. However, phylogenetic analyses of these hotspot regions (4807 bp in length) failed to discern the interrelationships of the closely related species and subclades of the genus (Figure S1) although this dataset could delimitate species units. Our previous study of *Orychophragmus* sequenced four cpDNA regions (*mat*K, *rbc*L, *trn*H-*psb*A, and *trn*L-F), and the total sequence alignment was around 3060 bp long (Hu H. et al., 2015). However, that cpDNA dataset failed to clarify species boundaries and construct interspecific relationships, whereas the nuclear ITS sequence dataset did despite only around 640 bp in length (Hu H. et al., 2015). Based on total plastome sequences or the hotspot mutation regions, the present study successfully differentiated all sampled species units although fewer individuals from different populations of each assumed species were used (Figure 3 and Figure S1). The study clearly suggests that the total plastomes or the hotspot mutation regions accumulated more mutations than few cpDNAs and were by far quite useful in the delimitation of boundaries of closely related species, as did nuclear ITS region (Hu H. et al., 2015). However, the interspecific relationships recovered by the plastomes differs from that from the nuclear ITS sequence variations (Figure 3). For example, *O. violaceus* is sister to *O. hupehensis* on the plastome tree while *O. violaceus* is closely related to *O. longisiliqus* on the ITS tree. In fact, none of the interspecific relationships inferred from the ITS sequence variations were confirmed by the plastome dataset (Figure 3). Only the accumulated mutations along the total plastome can delimit species boundaries (Figure 3), whereas the much shorter ITS fragments most likely had enough mutations to delineate species (Hu H. et al., 2015). In addition, we found that the estimated divergences between *Orychophragmus* and *Sinalliaria*, subclades, and species in the plastome phylogeny were also lower than those inferred from the ITS dataset. For example, the divergence time between *Orychophragmus* and *Sinalliaria* was estimated to occur around 13.92~9.16 Mya (Figure 4 and Figure S2), while that estimated on ITS sequence variations was about 20 Mya (Hu H. et al., 2015). The divergences within *Orychophragmus* were dated to between 5.9~3.25 to 0.96~0.59 Mya, in contrast to 7.7 to 2.7 Mya (Hu H. et al., 2015). This discrepancy was likely the result of different mutation rates between cpDNA and ITS. In addition, the estimations based on the hotspot mutation regions (Table S3) might be older than on the total plastomes when using the average substitution rate because of the accumulated divergences from these regions. It should be cautioned that all of the present estimates were conducted based on the average substitution rate or two secondary calibrations from the calibrated whole family plastome phylogeny (Hohmann et al., 2015). This second calibration might result in the unavoidable bias (Schenk, 2016). The generic assignment of the only relatively old fossil (*Thlaspi primaevum*) for the family and the age reliability are highly debated (Brenner, 1996; Beilstein et al., 2010; Franzke et al., 2016). All of these limitations restrict the direct and accurate calibration of the family and within-family lineages. However, the average plastome mutation rate (0.051952 ± 0.000537 × 10^−8^) fell well within the range rate recorded for chloroplast DNAs (Wolfe et al., 1987, 1989; Koch et al., 2000, 2001). Therefore, the estimated divergences presented here can still serve as a rough temporal framework for understanding the evolutionary diversification of the genus *Orychophragmus*, although further refined calibrations and estimates are highly needed.

Numerous studies (e.g., Ellstrand, 2014; Suh et al., 2015; Mallet et al., 2016; Novikova et al., 2016; Pease et al., 2016), reported inconsistent phylogenetic relationships within plants if constructed based on different genes or genomes, especially cpDNAs and ITS (Maddison, 1997; Morando et al., 2004; Arnold, 2006; Pollard et al., 2006). In most angiosperms, cpDNA has uniparental inheritance while the nuclear ITS is biparental (Hagemann, 2004; Petit et al., 2005; Wicke et al., 2011). Both incomplete lineage sorting and hybrid introgressions were suggested to explain such inconsistent relationships between ITS- and cpDNA-based phylogenies (Arnold, 2006). For example, *O. zhongtiaoshanus* probably experienced a strong genetic introgression from *O. diffusus*, or it propbably originated from hybridization between *O. diffusus* and *O. longisiliqus*. Similarly, gene flow might have occurred between *O. violaceus* and *O. hupehensis* and *O. longisiliqus*. It is also highly likely that incomplete lineage sorting occurred during the fast speciation of this genus that produced the current species, which resulted in the phylogenetic inconsistences between plastome and ITS datasets. During the fast radiative speciation of the genus *Arabidopsis*, ancestral polymorphisms at different loci were randomly fixed, and recent gene flow mediated the trans-specific introgressison of newly derived alleles (Novikova et al., 2016). Both incomplete lineage sorting and recent gene flow may have together resulted in the widespread inconsistences in gene trees and non-bifurcating speciation of *Arabidopsis*. Although, species divergences within *Orychophragmus*, as estimated on plastome or ITS (Hu H. et al., 2015), were older than those within *Arabidopsis* (Novikova et al., 2016), it is highly likely that non-bifurcating radiations with both incomplete lineage sorting and hybrid introgressions might have also occurred in the speciation history of *Orychophragmus*. Further studies based on nuclear genomic population data, as well as modeling tests, are needed to test the occurrences of both incomplete lineage sorting and trans-specific introgressions in *Orychophragmus*.

## Author contributions

HH and JL conceived and designed this study. HH and TZ collected the samples. HH, XL, and XG extracted total genomic DNA. HH and QH analyzed the data and wrote the manuscript. IA and JL revised the manuscript.

### Conflict of interest statement

The authors declare that the research was conducted in the absence of any commercial or financial relationships that could be construed as a potential conflict of interest.

## Supplementary material

Figure S1 **Maximum likelihood tree based on analyses of nine hotspot intergenic regions for 17 ***Orychophragmus*** individuals**. The ML tree is topologically congruent with the Bayesian consensus tree. Statistical support from maximum likelihood with different values were shown as different line forms. The different taxa of *Orychophragmus* and *Sinalliaria* are marked by different colors.

Figure S2 **Divergence time estimates using two secondary points based on the whole plastid genome sequences**. Divergence times of species based on uncorrelated relaxed clock method, using two calibrations (23.5 million years ago (Ma) between the *Arabidopsis* clade vs. the sister clade and 20.85 Ma between the *Lobularia* subclade vs. the sister subclade) calculated by BEAST program over the whole chloroplast genome sequences. The legend describes the divergence time in million years, and the gray boxes represent the 95% highest probability density of divergence times.

Table S1 **List of samples used in this chloroplast phylogenomic analyses**.

Table S2 **The best-fit model of each plastome section chosen by Jmodeltest using Bayesian Information Criterion (BIC) and Akaike Information Criterion (AIC)**.

Table S3 **Relaxed clock age estimates obtained with BEAST for the nodes of interest shown and numbered in Figure 4**. Ma, million years ago.

## References

[B1] Al-Shehbaz I. A. Yang G. (2000). A revision of the Chinese endemic *Orychophragmus* (Brassicaceae). Novon, 10, 349–353. 10.2307/3392983

[B2] Arbiza L. Patricio M. Dopazo H. Posada D. (2011). Genome-wide heterogeneity of nucleotide substitution model fit. Genome Biol. Evol. 3, 896–908. 10.1093/gbe/evr080 21824869 PMC3175760

[B3] Arnold M. L. (2006). Evolution Through Genetic Exchange. Oxford: Oxford University Press.

[B4] Beilstein M. A. Nagalingum N. S. Clements M. D. Manchester S. R. Mathews S. (2010). Dated molecular phylogenies indicate a Miocene origin for *Arabidopsis thaliana*. Proc. Natl. Acad. Sci. U.S.A. 107, 18724–18728. 10.1073/pnas.0909766107 20921408 PMC2973009

[B5] Bock R. (2007). Structure, function, and inheritance of plastid genomes, in Cell and Molecular Biology of Plastids, ed Bock R. (Berlin; Heidelberg: Springer), 29–63. 10.1007/4735_2007_0223

[B6] Bouckaert R. Heled J. Kühnert D. Vaughan T. Wu C. H. Xie D.. (2014). BEAST 2: a software platform for Bayesian evolutionary analysis. PLoS Comput. Biol. 10:e1003537. 10.1371/journal.pcbi.1003537 24722319 PMC3985171

[B7] Brenner G. J. (1996). Evidence for the earliest stage of angiosperm pollen evolution: a paleoequatorial section from Israel, in Flowering Plant Origin, Evolution and Phylogeny, eds Taylor D. W. Hickey L. J. (New York, NY: Springer US), 91–115. 10.1007/978-0-585-23095-5_5

[B8] Carbonell-Caballero J. Alonso R. Ibañez V. Terol J. Talon M. Dopazo J. (2015). A phylogenetic analysis of 34 chloroplast genomes elucidates the relationships between wild and domestic species within the genus *Citrus*. Mol. Biol. Evol. 32, 2015–2035. 10.1093/molbev/msv082 25873589 PMC4833069

[B9] Corriveau J. L. Coleman A. W. (1988). Rapid screening method to detect potential biparental inheritance of plastid DNA and results for over 200 angiosperm species. Am. J. Bot. 75, 1443–1458. 10.2307/2444695

[B10] Darriba D. Taboada G. L. Doallo R. Posada D. (2012). jModelTest 2: more models, new heuristics and parallel computing. Nat. Methods 9, 772–772. 10.1038/nmeth.2109 22847109 PMC4594756

[B11] Doyle J. J. (1987). A rapid DNA isolation procedure for small quantities of fresh leaf tissue. Phytochem. Bull. 19, 11–15.

[B12] Drummond A. J. Ho S. Y. Phillips M. J. Rambaut A. (2006). Relaxed phylogenetics and dating with confidence. PLoS Biol. 4:e88. 10.1371/journal.pbio.0040088 16683862 PMC1395354

[B13] Ellstrand N. C. (2014). Is gene flow the most important evolutionary force in plants? Am. J. Bot. 101, 737–753. 10.3732/ajb.1400024 24752890

[B14] Franzke A. Koch M. A. Mummenhoff K. (2016). Turnip time travels: age estimates in Brassicaceae. Trends Plant Sci. 21, 554–561. 10.1016/j.tplants.2016.01.024 26917156

[B15] Hagemann R. (2004). The sexual inheritance of plant organelles, in Molecular Biology and Biotechnology of Plant Organelles, eds Daniell H. Chase C. (Springer Netherlands), 93–113. 10.1007/978-1-4020-3166-3_4

[B16] Hohmann N. Wolf E. M. Lysak M. A. Koch M. A. (2015). A time-calibrated road map of Brassicaceae species radiation and evolutionary history. Plant Cell 27, 2770–2784. 10.1105/tpc.15.00482 26410304 PMC4682323

[B17] Hu H. Al-Shehbaz I. A. Sun Y. Hao G. Wang Q. Liu J. (2015). Species delimitation in *Orychophragmus* (Brassicaceae) based on chloroplast and nuclear DNA barcodes. Taxon 64, 714–726. 10.12705/644.4

[B18] Hu S. L. Sablok G. Wang B. Qu D. Barbaro E. Viola R.. (2015). Plastome organization and evolution of chloroplast genes in Cardamine species adapted to contrasting habitats. BMC Genomics 16:306. 10.1186/s12864-015-1498-0 25887666 PMC4446112

[B19] Huang D. I. Cronk Q. C. (2015). Plann: a command-line application for annotating plastome sequences. Appl. Plant Sci. 3, 1–3. 10.3732/apps.1500026 26312193 PMC4542940

[B20] Huang H. Shi C. Liu Y. Mao S. Y. Gao L. Z. (2014). Thirteen Camellia chloroplast genome sequences determined by high-throughput sequencing: genome structure and phylogenetic relationships. BMC Evol. Biol. 14:151. 10.1186/1471-2148-14-151 25001059 PMC4105164

[B21] Jansen R. K. Cai Z. Raubeson L. A. Daniell H. Leebens-Mack J. Müller K. F.. (2007). Analysis of 81 genes from 64 plastid genomes resolves relationships in angiosperms and identifies genome-scale evolutionary patterns. Proc. Natl. Acad. Sci. U.S.A. 104, 19369–19374. 10.1073/pnas.0709121104 18048330 PMC2148296

[B22] Jansen R. K. Ruhlman T. A. (2012). Plasti genomes of seed plants, in Genomics of Chloroplasts and Mitochondria, eds Bock R. Knoop V. (Springer Netherlands), 103–126. 10.1007/978-94-007-2920-9_5

[B23] Katoh K. Standley D. M. (2013). MAFFT multiple sequence alignment software version 7: improvements in performance and usability. Mol. Biol. Evol. 30, 772–780. 10.1093/molbev/mst010 23329690 PMC3603318

[B24] Kearse M. Moir R. Wilson A. Stones-Havas S. Cheung M. Sturrock S.. (2012). Geneious Basic: an integrated and extendable desktop software platform for the organization and analysis of sequence data. Bioinformatics 28, 1647–1649. 10.1093/bioinformatics/bts199 22543367 PMC3371832

[B25] Koch M. A. Haubold B. Mitchell-Olds T. (2000). Comparative evolutionary analysis of chalcone synthase and alcohol dehydrogenase loci in *Arabidopsis*, Arabis, and related genera (Brassicaceae). Mol. Biol. Evol. 17, 1483–1498. 10.1093/oxfordjournals.molbev.a026248 11018155

[B26] Koch M. Haubold B. Mitchell-Olds T. (2001). Molecular systematics of the Brassicaceae: evidence from coding plastidic matK and nuclear Chs sequences. Am. J. Bot. 88, 534–544. 10.2307/2657117 11250830

[B27] Lamichhaney S. Berglund J. Almén M. S. Maqbool K. Grabherr M. Martinez-Barrio A.. (2015). Evolution of Darwin's finches and their beaks revealed by genome sequencing. Nature 518, 314–317. 10.1038/nature14181 25686609

[B28] Li H. Handsaker B. Wysoker A. Fennell T. Ruan J. Homer N.. (2009). The sequence alignment/map format and SAMtools. Bioinformatics 25, 2078–2079. 10.1093/bioinformatics/btp352 19505943 PMC2723002

[B29] Li H. Durbin R. (2009). Fast and accurate short read alignment with Burrows–Wheeler transform. Bioinformatics 25, 1754–1760. 10.1093/bioinformatics/btp324 19451168 PMC2705234

[B30] Lohse M. Drechsel O. Kahlau S. Bock R. (2013). OrganellarGenomeDRAW—a suite of tools for generating physical maps of plastid and mitochondrial genomes and visualizing expression data sets. Nucleic Acids Res. 41, 75–81. 10.1093/nar/gkt289 23609545 PMC3692101

[B31] Maddison W. P. (1997). Gene trees in species trees. Syst. Biol. 46, 523–536. 10.1093/sysbio/46.3.523

[B32] Mallet J. Besansky N. Hahn M. W. (2016). How reticulated are species? Bioessays 38, 140–149. 10.1002/bies.201500149 26709836 PMC4813508

[B33] Mayor C. Brudno M. Schwartz J. R. Poliakov A. Rubin E. M. Frazer K. A.. (2000). VISTA: visualizing global DNA sequence alignments of arbitrary length. Bioinformatics 16, 1046–1047. 10.1093/bioinformatics/16.11.1046 11159318

[B34] Moore M. J. Soltis P. S. Bell C. D. Burleigh J. G. Soltis D. E. (2010). Phylogenetic analysis of 83 plastid genes further resolves the early diversification of eudicots. Proc. Natl. Acad. Sci. U.S.A. 107, 4623–4628. 10.1073/pnas.0907801107 20176954 PMC2842043

[B35] Morando M. Avila L. J. Baker J. Sites J. W. (2004). Phylogeny and phylogeography of the Liolaemus darwinii complex (Squamata: Liolaemidae): evidence for introgression and incomplete lineage sorting. Evolution 58, 842–859. 10.1111/j.0014-3820.2004.tb00416.x 15154559

[B36] Nock C. J. Waters D. L. Edwards M. A. Bowen S. G. Rice N. Cordeiro G. M.. (2011). Chloroplast genome sequences from total DNA for plant identification. Plant Biotechnol. J. 9, 328–333. 10.1111/j.1467-7652.2010.00558.x 20796245

[B37] Novikova P. Y. Hohmann N. Nizhynska V. Tsuchimatsu T. Ali J. Muir G.. (2016). Sequencing of the genus *Arabidopsis* identifies a complex history of nonbifurcating speciation and abundant trans-specific polymorphism. Nat. Genet. 48, 1077–1082. 10.1038/ng.3617 27428747

[B38] Palmer J. D. (1991). Plastid chromosomes: structure and evolution. Mol. Biol. Plastids 7, 5–53. 10.1016/B978-0-12-715007-9.50009-8

[B39] Parks M. Cronn R. Liston A. (2009). Increasing phylogenetic resolution at low taxonomic levels using massively parallel sequencing of chloroplast genomes. BMC Biol. 7:84. 10.1186/1741-7007-7-84 19954512 PMC2793254

[B40] Pease J. B. Haak D. C. Hahn M. W. Moyle L. C. (2016). Phylogenomics reveals three sources of adaptive variation during a rapid radiation. PLoS Biol. 14:e1002379. 10.1371/journal.pbio.1002379 26871574 PMC4752443

[B41] Petit R. J. Duminil J. Fineschi S. Hampe A. Salvini D. Vendramin G. G. (2005). Invited review: comparative organization of chloroplast, mitochondrial and nuclear diversity in plant populations. Mol. Ecol. 14, 689–701. 10.1111/j.1365-294X.2004.02410.x 15723661

[B42] Pollard D. A. Iyer V. N. Moses A. M. Eisen M. B. (2006). Widespread discordance of gene trees with species tree in *Drosophila*: evidence for incomplete lineage sorting. PLoS Genet. 2:e173. 10.1371/journal.pgen.0020173 17132051 PMC1626107

[B43] Posada D. (2008). jModelTest: phylogenetic model averaging. Mol. Biol. Evol. 25, 1253–1256. 10.1093/molbev/msn083 18397919

[B44] Rambaut A. Suchard M. Xie D. Drummond A. (2014). Tracer v. 1.6. Institute of Evolutionary Biology, University of Edinburgh. Available online at: http://beast.bio.ed.ac.uk/Tracer

[B45] Raubeson L. A. Jansen R. K. (2005). 4 Chloroplast genomes of plants, in Plant Diversity and Evolution: Genotypic and Phenotypic Variation in Higher Plants, ed Henry R. J. (Wallingford, CT: CAB International), 45–68.

[B46] Ronquist F. Huelsenbeck J. P. (2003). MrBayes 3: Bayesian phylogenetic inference under mixed models. Bioinformatics 19, 1572–1574. 10.1093/bioinformatics/btg180 12912839

[B47] Ronquist F. Teslenko M. van der Mark P. Ayres D. L. Darling A. Höhna S.. (2012). MrBayes 3.2: efficient Bayesian phylogenetic inference and model choice across a large model space. Syst. Biol. 61, 539–542. 10.1093/sysbio/sys029 22357727 PMC3329765

[B48] Schenk J. J. (2016). Consequences of secondary calibrations on divergence time estimates. PLoS ONE 11:e0148228. 10.1371/journal.pone.0148228 26824760 PMC4732660

[B49] Schluter D. (2000). The Ecology of Adaptive Radiation. Oxford: Oxford University Press.

[B50] Stamatakis A. (2014). RAxML version 8: a tool for phylogenetic analysis and post-analysis of large phylogenies. Bioinformatics 30, 1312–1313. 10.1093/bioinformatics/btu033 24451623 PMC3998144

[B51] Suh A. Smeds L. Ellegren H. (2015). The dynamics of incomplete lineage sorting across the ancient adaptive radiation of neoavian birds. PLoS Biol. 13:e1002224. 10.1371/journal.pbio.1002224 26284513 PMC4540587

[B52] Sun L. Fang L. Zhang Z. Chang X. Penny D. Zhong B. (2016). Chloroplast phylogenomic inference of green algae relationships. Sci. Rep. 6:20528. 10.1038/srep20528 26846729 PMC4742797

[B53] Sun L. Yang W. W. Zhang Y. Jin X. F. (2012). The seedling morphology of *Orychophragmus* and relatives (Cruciferae) and its taxonomic significance. J. Hangzhou Normal Univ. 4, 337–341.

[B54] Sun X. Q. Pang H. Guo J. L. Peng B. Bai M. M. Hang Y. Y. (2011). Fatty acid analysis of the seed oil in a germplasm collection of 94 species in 58 genera of Brassicaceae. Chem. Indus. Forest Prod. 31, 46–54.

[B55] Tamura K. Stecher G. Peterson D. Filipski A. Kumar S. (2013). MEGA6: molecular evolutionary genetics analysis version 6.0. Mol. Biol. Evol. 30, 2725–2729. 10.1093/molbev/mst197 24132122 PMC3840312

[B56] Tan Z. M. Xu J. M. Zhao B. X. Zhang X. L. (1998). New taxa of *Orychophragmus* (Cruciferae) from China. *Acta Phytotax*. Sin. 36, 544–548.

[B57] Wicke S. Schneeweiss G. M. Müller K. F. Quandt D. (2011). The evolution of the plastid chromosome in land plants: gene content, gene order, gene function. Plant Mol. Biol. 76, 273–297. 10.1007/s11103-011-9762-4 21424877 PMC3104136

[B58] Wolfe K. H. Li W. H. Sharp P. M. (1987). Rates of nucleotide substitution vary greatly among plant mitochondrial, chloroplast, and nuclear DNAs. Proc. Natl. Acad. Sci. U.S.A. 84, 9054–9058. 10.1073/pnas.84.24.9054 3480529 PMC299690

[B59] Wolfe K. H. Sharp P. M. Li W. H. (1989). Rates of synonymous substitution in plant nuclear genes. J. Mol. Evol. 29, 208–211. 10.1007/BF02100204

[B60] Wu F. H. Chan M. T. Liao D. C. Hsu C. T. Lee Y. W. Daniell H.. (2010). Complete chloroplast genome of *Oncidium* Gower Ramsey and evaluation of molecular markers for identification and breeding in Oncidiinae. BMC Plant Biol. 10:68. 10.1186/1471-2229-10-68 20398375 PMC3095342

[B61] Wu J. Zhao Z. (2003). A new species of *Orychophragmus* (Cruciferae) in the Three-Gorge Reservoir area, China. Wuhan Bot. Res. 21, 487–488.

[B62] Yang J. B. Tang M. Li H. T. Zhang Z. R. Li D. Z. (2013). Complete chloroplast genome of the genus *Cymbidium*: lights into the species identification, phylogenetic implications and population genetic analyses. BMC Evol. Biol. 13:84. 10.1186/1471-2148-13-84 23597078 PMC3644226

[B63] Yoder J. B. Briskine R. Mudge J. Farmer A. Paape T. Steele K.. (2013). Phylogenetic signal variation in the genomes of Medicago (Fabaceae). Syst. Biol. 62, 424–438. 10.1093/sysbio/syt009 23417680

[B64] Zeng T. T. Hu H. Guo X. Y. Hu Q. J. (2016). The complete chloroplast genomes of two *Sinalliaria* species and species delimitation (Brassicaceae). Conserv. Genet. Resour. 8, 379–381. 10.1007/s12686-016-0563-6

[B65] Zerbino D. R. Birney E. (2008). Velvet: algorithms for de novo short read assembly using de Bruijn graphs. Genome Res. 18, 821–829. 10.1101/gr.074492.107 18349386 PMC2336801

[B66] Zhang Q. Liu Y. (2003). Examination of the cytoplasmic DNA in male reproductive cells to determine the potential for cytoplasmic inheritance in 295 angiosperm species. Plant Cell Physiol. 44, 941–951. 10.1093/pcp/pcg121 14519776

[B67] Zhong B. Deusch O. Goremykin V. V. Penny D. Biggs P. J. Atherton R. A.. (2011). Systematic error in seed plant phylogenomics. Genome Biol. Evol. 3, 1340–1348. 10.1093/gbe/evr105 22016337 PMC3237385

[B68] Zhou T. Y. (1987). Orychophragmus, in Flora Reipublicae Popularis Sinicae, Vol. 33, ed Zhou T. Y. (Beijing: Science Press), 40–43.

[B69] Zhou T. Y. Lu L. L. Yang G. Al-Shehbaz I. A. (2001). Orychophragmus Bunge, in Flora of China, Vol. 8, eds Wu Z. Y. Raven P. H. (Beijing; St. Louis, MO: Missouri Botanical Garden Press; Science Press), 29–31.

[B70] Zhou Y. Y. Zhang H. W. Hu J. Q. Jin X. F. (2014). *Sinalliaria*, a new genus of Brassicaceae from eastern China, based on morphological and molecular data. Phytotaxa 186, 188–198. 10.11646/phytotaxa.186.4.2

