# Supplementary figures and images for: Species Delimitation and Interspecific Relationships of the Genus Orychophragmus (Brassicaceae) Inferred from Whole Chloroplast Genomes
Source: Front Plant Sci. 2016 Dec 6;7:1826. doi: 10.3389/fpls.2016.01826 (PMC5138468; doi:10.3389/fpls.2016.01826)

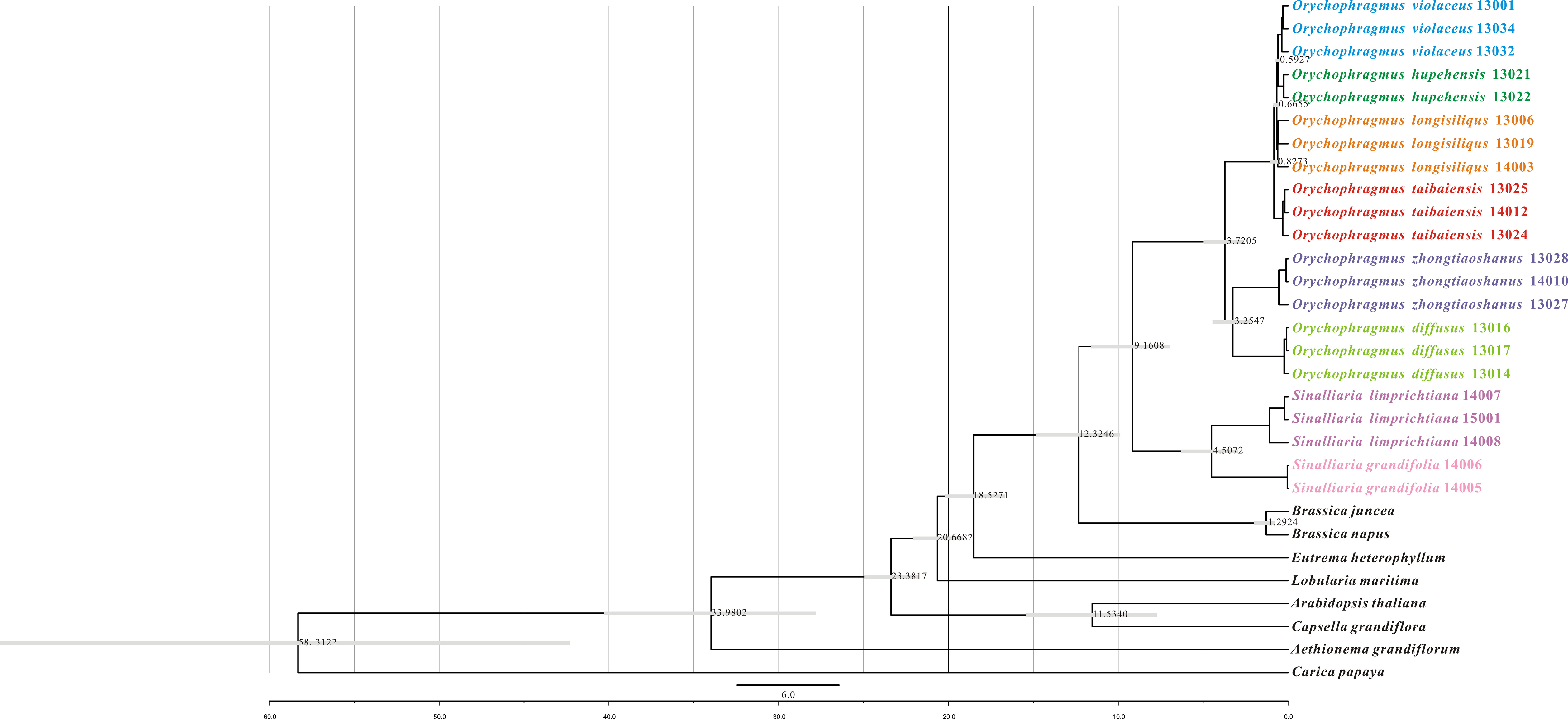

Supplement: Figure S2 — Divergence time estimates using two secondary points based on the whole plastid genome sequences. Divergence times of species based on uncorrelated relaxed clock method, using two calibrations (23.5 million years ago (Ma) between the Arabidopsis clade vs. the sister clade and 20.85 Ma between the Lobularia subclade vs. the sister subclade) calculated by BEAST program over the whole chloroplast genome sequences. The legend describes the divergence time in million years, and the gray boxes represent the 95% highest probability density of divergence times. [file Image2.JPEG]
