# Supplementary material for: Species Delimitation and Interspecific Relationships of the Genus Orychophragmus (Brassicaceae) Inferred from Whole Chloroplast Genomes
Source: Front Plant Sci. 2016 Dec 6;7:1826. doi: 10.3389/fpls.2016.01826 (PMC5138468; doi:10.3389/fpls.2016.01826)
Supplement: Table S1 — List of samples used in this chloroplast phylogenomic analyses. [file DataSheet1.docx]

**Table S1. List of samples used in this chloroplast phylogenomic analyses.**

| **Species** | **Individuals** | **No. of populations** | **Lat. (°E), Long. (°N)** | **Alt. (m)** | **GenBank accesion** |
| --- | --- | --- | --- | --- | --- |
| *Orychophragmus violaceus* | 3 | 13001 | 113.70609, 034.71227 | 130 | KX364399 (13001) |
|  |  | 13034 | 117.02884, 036.63781 | 190 |  |
|  |  | 13032 | 113.56596, 035.61166 | 393 |  |
| *Orychophragmus longisiliqus* | 3 | 13006 | 110.68559, 031.74689 | 916 | KX756549 (13006) |
|  |  | 13019 | 113.94205, 031.84963 | 232 |  |
|  |  | 14003 | 119.16523, 030.01235 | 214 |  |
| *Orychophragmus zhongtiaoshanus* | 3 | 14010 | 111.05296, 034.94369 | 709 | KX756547(14010) |
|  |  | 13027 | 110.07903, 034.51179 | 750 |  |
|  |  | 13028 | 110.44849, 034.84198 | 556 |  |
| *Orychophragmus diffusus* | 3 | 13017 | 121.18791, 031.09589 | 128 | KX756548(13017) |
|  |  | 13014 | 120.20732, 030.43667 | 251 |  |
|  |  | 13016 | 121.17908, 031.08108 | 30 |  |
| *Orychophragmus hupehensis* | 2 | 13022 | 111.01426, 032.42040 | 922 | KX756551(13022) |
|  |  | 13021 | 111.05944, 032.44725 | 430 |  |
| *Orychophragmus taibaiensis* | 3 | 14012 | 107.70551, 034.08924 | 1133 | KX756550(14012) |
|  |  | 13024 | 107.71554, 034.09716 | 1128 |  |
|  |  | 13025 | 107.62776, 034.04383 | 938 |  |
| *Sinalliaria limprichtiana* | 3 | 14007/14008/15001 |  |  | KX342848 (14007) |
| *Sinalliaria grandifolia* | 2 | 14005/14006 |  |  | KX342847 (14005) |
| *Carica papaya* |  |  |  |  | EU431223 |
| *Brassica napus* |  |  |  |  | GQ861354 |
| *Brassica juncea* |  |  |  |  | KT581449 |
| *Eutrema heterophyllum* |  |  |  |  | KT270358 |
| *Lobularia maritima* |  |  |  |  | NC_009274 |
| *Capsella grandiflora* |  |  |  |  | KR029092 |
| *Arabidopsis thaliana* |  |  |  |  | AP000423 |
| *Aethionema grandiflorum* |  |  |  |  | NC_009266 |
| *Eutrema yunnanense* |  |  |  |  | KT270357 |
| *Schrenkiella parvula* |  |  |  |  | KT222186 |
| *Eutrema salsugineum* |  |  |  |  | KR584659 |
| *Pachycladon fastigiatum* |  |  |  |  | NC_029768 |
| *Eutrema botschantzevii* |  |  |  |  | KT962847 |
| *Eutrema halophilum* |  |  |  |  | KT962846 |
| *Isatis tinctoria* |  |  |  |  | KT591187 |
| *Capsella rubella* |  |  |  |  | KR029093 |
| *Brassica oleracea* var. *capitata* |  |  |  |  | KR233156 |
| *Raphanus sativus* |  |  |  |  | KJ716483 |
| *Pachycladon cheesemanii* |  |  |  |  | JQ806762 |
| *Pachycladon enysii* |  |  |  |  | JX205495 |
| *Brassica rapa* subsp. *pekinensis* |  |  |  |  | NC_015139 |
| *Aethionema cordifolium* |  |  |  |  | NC_009265 |
| *Arabis hirsuta* |  |  |  |  | NC_009268 |
| *Draba nemorosa* |  |  |  |  | NC_009272 |
| *Crucihimalaya wallichii* |  |  |  |  | NC_009271 |
| *Lepidium virginicum* |  |  |  |  | NC_009273 |
| *Barbarea verna* |  |  |  |  | NC_009269 |
| *Capsella bursa-pastoris* |  |  |  |  | NC_009270 |

**Table S2. The best-fit model of each plastome section chosen by Jmodeltest using Bayesian Information Criterion (BIC) and Akaike Information Criterion (AIC).**

| Region | Best-fit model by BIC | Best-fit model by AIC |
| --- | --- | --- |
| Full-length sequence | TVM+I+G | TVM+I+G |
| IRs | TPM1uf+I+G | TVM+I+G |
| LSC | TVM+I+G | GTR+I+G |
| SSC | TVM+I+G | GTR+I+G |
| Coding region | TVM+I+G | TVM+I+G |
| Non-coding region | TVM+I+G | TVM+I+G |
| Nine divergence hotspot regions | TPM1uf+ G | TVM+G |

**Table S3. Relaxed clock age estimates obtained with BEAST for the nodes of interest shown and numbered in Figure 4. Ma: million years ago.**

| Node | Node description | BEAST (Ma) analyses of the whole plastomes | | BEAST (Ma) analyses of nine divergence hotspot regions | |
| --- | --- | --- | --- | --- | --- |
|  |  | average substitution rate | two calibration points | average substitution rate | two calibration points |
| 1 | Crown of *Orychophragmus* | 5.9 (4.3~7.6) | 3.7 (2.6~5.0) | 19.3 (9.4~32.9) | 4.4 (2.4~6.6) |
| 2 | Crown of *Sinalliaria* | 7.0 (4.7~9.4) | 4.5 (2.9~6.3) | 18.3 (7.7~31.9) | 4.3 (2.1~7.1) |
| 3 | Split: *Orychophragmus* and *Sinalliaria* | 13.9 (10.8~17.4) | 9.2 (6.9~11.6) | 37.1 (21.0~54.3) | 9.5 (6.0~13.1) |
